# Refining Postoperative Intensive Care Triage After Anatomical Lung Resection: A Retrospective Cohort Study of Perioperative Reassessment

**DOI:** 10.3390/jcm15135043

**Published:** 2026-06-28

**Authors:** Dilara Tüfek Öztan, Hacer Boztepe Yeşilçay, Şencan Akdağ, Mustafa Ay, Şule Asri

**Affiliations:** 1 Department of Intensive Care, Antalya Training and Research Hospital, University of Health Sciences Türkiye, 07100 Antalya, Türkiye; mustafaayidil@hotmail.com (M.A.); drsuleasri@gmail.com (Ş.A.); 2 Department of Thoracic Surgery, Antalya Training and Research Hospital, University of Health Sciences Türkiye, 07100 Antalya, Türkiye; drhacer83@hotmail.com (H.B.Y.); sencanakdag@hotmail.com (Ş.A.)

**Keywords:** non-small cell lung cancer, anatomical lung resection, intensive care unit admission, ICU triage, perioperative reassessment, risk prediction, postoperative care

## Abstract

**Background/Objectives:** Postoperative intensive care unit (ICU) disposition after anatomical lung resection is usually planned preoperatively, but the final care pathway may be substantially influenced by intraoperative events. We evaluated actual postoperative ICU admission among patients with a documented preoperative ICU monitoring recommendation and compared preoperative-only and perioperative triage approaches. **Methods:** In this retrospective single-centre cohort study, 1060 adults undergoing elective anatomical lung resection for non-small cell lung cancer (NSCLC) between January 2019 and December 2025 were screened; 159 patients with a documented preoperative ICU monitoring recommendation constituted the analytical cohort. A clinically pre-specified primary perioperative model incorporating operative duration, intraoperative complication, chronic obstructive pulmonary disease (COPD), and pre-existing arrhythmia was compared with a preoperative-only model and with an exploratory perioperative ICU triage score. **Results:** Actual postoperative ICU admission occurred in 45 patients (28.3%). Operative duration (adjusted odds ratio [OR] 1.012 per minute; 95% confidence interval [CI], 1.005–1.018; *p* < 0.001) and intraoperative complication (adjusted OR 15.002; 95% CI, 3.738–60.210; *p* < 0.001) were significantly associated with actual postoperative ICU admission. The primary perioperative model achieved an AUC of 0.802 (95% CI, 0.717–0.876), compared with 0.759 for the exploratory perioperative triage score and 0.665 for the preoperative-only model. **Conclusions:** Fewer than one-third of patients with a documented preoperative ICU monitoring recommendation underwent actual postoperative ICU admission. In this selected cohort, perioperative reassessment incorporating intraoperative information showed higher apparent discriminative performance than the preoperative-only approach while the exploratory score showed intermediate, hypothesis-generating performance. Because the outcome reflected observed institutional ICU disposition rather than independently adjudicated ICU-level care requirement, prospective multicentre validation using predefined ICU admission criteria is required before clinical implementation.

## 1. Introduction

Non-small cell lung cancer (NSCLC) accounts for approximately 85% of all lung cancer diagnoses and represents one of the leading causes of cancer-related mortality worldwide [1]. In patients with resectable disease, anatomical lung resection—encompassing lobectomy, segmentectomy, bilobectomy, and pneumonectomy—constitutes the cornerstone of curative-intent treatment [2,3,4]. Advances in minimally invasive surgical approaches and refinements in perioperative management have expanded operative candidacy to older patients and those carrying a substantial comorbidity burden [5]. Accordingly, accurate perioperative risk stratification and determination of the appropriate level of postoperative monitoring have become increasingly central to clinical decision-making in thoracic oncological surgery [6].

Identifying patients who may require postoperative intensive care unit (ICU) monitoring after anatomical lung resection has important implications for patient safety and the efficient allocation of finite ICU resources [7]. In current clinical practice, this planning decision is typically made at the time of the preoperative anaesthesia assessment, during which pulmonary function parameters, cardiac evaluation findings, comorbidity burden, functional status, American Society of Anesthesiologists (ASA) physical status classification, and anticipated surgical risk are considered in aggregate [8]. Established risk stratification frameworks—including the European Respiratory Society/European Society of Thoracic Surgeons (ERS/ESTS) physiological assessment guidelines [9], the Thoracic Surgery Scoring System (Thoracoscore) [10], and the Society of Thoracic Surgeons (STS) risk models [11]—provide structured approaches to perioperative risk appraisal in lung resection candidates. However, these tools were developed primarily to assess surgical candidacy, postoperative mortality risk, or major morbidity, and were not specifically designed to guide observed postoperative ICU disposition or final postoperative triage decisions [12].

While preoperative evaluation constitutes the foundation of perioperative planning, it is inherently limited in its capacity to anticipate the dynamic physiological and procedural events that arise during surgery and anaesthesia. Prior evidence has demonstrated that preoperative assessment alone may fail to identify a substantial proportion of patients who ultimately met predefined postoperative critical care criteria, with sensitivity as low as 53.3% reported in a prospective cohort of elective pulmonary resection candidates [13]. Complementarily, Kim and colleagues retrospectively analysed 340 candidates for preoperative ICU reservation and identified greater intraoperative haemorrhage and thoracotomy access as independent determinants of postoperative critical care admission according to their study criteria [12]. Collectively, these findings indicate that postoperative care planning may benefit from structured reassessment at the conclusion of surgery, informed by the observed intraoperative course.

Despite this accumulating evidence, important gaps persist. The perioperative variables associated with actual postoperative ICU admission and the comparative discriminative performance of different triage approaches have not been systematically evaluated in a well-defined cohort of NSCLC patients undergoing anatomical lung resection with a documented preoperative recommendation for ICU monitoring. Discordance between anticipated and observed postoperative care levels may contribute to inefficient ICU resource use and delayed recognition of patients who may require escalation to enhanced postoperative monitoring, particularly when final disposition decisions rely primarily on information available before surgery.

The primary aim of this study was to evaluate the rate of actual postoperative ICU admission and to examine the perioperative variables associated with this outcome in NSCLC patients who underwent anatomical lung resection and had a documented preoperative recommendation for ICU monitoring. As a secondary exploratory aim, we sought to develop a pragmatic perioperative ICU triage score incorporating both preoperative and intraoperative variables, and to compare its discriminative performance with that of a preoperative-only multivariable model and the primary perioperative multivariable model. Accordingly, the study focused on observed postoperative ICU admission within a selected institutional cohort rather than on an externally adjudicated definition of ICU-level care requirement.

## 2. Materials and Methods

### 2.1. Study Design and Setting

This retrospective, single-centre, observational cohort study was conducted at the University of Health Sciences Türkiye, Antalya Training and Research Hospital, a tertiary thoracic surgery referral centre in Antalya, Türkiye. The study was approved by the Antalya Training and Research Hospital Medical Research Scientific Ethics Committee (Decision No: 21/8; Protocol No: 2025-481; Date: 11 December 2025). The requirement for individual informed consent was waived owing to the retrospective observational design of the study. Reporting followed the Strengthening the Reporting of Observational Studies in Epidemiology (STROBE) statement [14], and the predictive model development and internal validation components were reported in accordance with the TRIPOD statement [15].

### 2.2. Study Population and Cohort Selection

The analytical cohort was restricted to patients with histopathologically confirmed primary NSCLC who underwent elective anatomical lung resection and had a documented preoperative recommendation for postoperative ICU monitoring. This recommendation was defined as a written entry recorded by the attending anaesthesiologist on the routine preoperative anaesthesia assessment form during the preoperative anaesthesia clinic evaluation, reflecting individualised clinical judgement regarding anticipated postoperative monitoring needs rather than the output of a standardised scoring algorithm. This clinician-based assessment approach may have introduced inter-individual and temporal variability into cohort selection, as institutional thresholds and perioperative pathways may have varied across the six-year study period. Furthermore, ICU bed availability may have influenced observed postoperative ICU admission decisions alongside clinical considerations.

The screened study population comprised all patients who underwent elective anatomical lung resection for primary NSCLC during the study period after application of the predefined disease-specific eligibility criteria. Therefore, the flow diagram begins with this disease-specific screened cohort (*n* = 1060) rather than the broader thoracic surgery database. Within this screened population, 159 patients had a documented preoperative recommendation for postoperative ICU monitoring and were therefore included in the analytical cohort. The remaining 901 patients did not have such a documented recommendation and were not included in the analytical cohort.

Thus, the final analytical cohort comprised 159 patients, corresponding to 15.0% of the screened population. This design intentionally focused on patients with a documented preoperative recommendation for ICU monitoring; however, it also limits generalisability to the broader population of patients undergoing anatomical lung resection. Because the analytical database was constructed specifically for patients with a documented preoperative ICU monitoring recommendation, detailed baseline and perioperative characteristics of patients outside the analytical cohort were not collected for the present study and were therefore not available for comparative analysis.

#### 2.2.1. Inclusion Criteria

Patients were eligible for inclusion if they met all of the following: age ≥18 years; histopathologically confirmed primary NSCLC on final pathological examination; elective curative-intent anatomical lung resection, including segmentectomy, lobectomy, bilobectomy, or pneumonectomy; a written preoperative recommendation for postoperative ICU monitoring recorded by the attending anaesthesiologist on the routine preoperative anaesthesia assessment form; and availability of all clinical, perioperative, and 30-day outcome variables required for the planned analyses.

#### 2.2.2. Exclusion Criteria

Patients were excluded if any of the following criteria were present: surgical procedure other than curative-intent anatomical resection, including wedge resection, exploratory thoracotomy, or diagnostic biopsy; resection performed for metastatic disease or a non-primary lung malignancy; resection for a neuroendocrine lung tumour; receipt of preoperative neoadjuvant chemotherapy, immunotherapy, and/or radiotherapy, as these patients were considered to have a distinct perioperative risk profile related to treatment-associated physiological alterations; documented active infection or decompensated comorbidity at the time of surgery; or missing data for key clinical, perioperative, or outcome variables.

### 2.3. Data Collection and Variables

Clinical, laboratory, operative, and postoperative data were retrospectively obtained from the institutional electronic medical record system, operating room anaesthesia records, post-anaesthesia care unit (PACU) observation records, ICU records, and archived patient files.

Preoperative variables included age, sex, smoking exposure (pack-years), ASA physical status classification, Eastern Cooperative Oncology Group (ECOG) performance status, Modified Frailty Index-11 (mFI-11) [16], Charlson Comorbidity Index (CCI) [17], preoperative peripheral oxygen saturation (SpO_2_), and C-reactive protein (CRP). Recorded comorbidities included hypertension, diabetes mellitus, COPD, coronary artery disease, pre-existing arrhythmia, valvular heart disease, previous coronary angioplasty or stent implantation, previous cerebrovascular event, and heart failure.

Pulmonary function assessment included forced expiratory volume in one second expressed as a percentage of the predicted value (FEV_1_%), forced vital capacity expressed as a percentage of the predicted value (FVC%), and the FEV_1_/FVC ratio expressed as a percentage (FEV_1_/FVC%), obtained from spirometry performed within 15 days before surgery. Echocardiographic assessment was categorised as normal, pathological, or not clinically indicated following cardiovascular evaluation; the latter category did not represent missing data.

Intraoperative variables included the type of anatomical resection, surgical approach [video-assisted thoracoscopic surgery (VATS), robot-assisted thoracoscopic surgery (RATS), or open thoracotomy], operative duration in minutes, estimated blood loss in millilitres, presence of an intraoperative complication, and vasopressor use. Intraoperative complication was recorded as a binary variable and defined as any clinically significant adverse event requiring active intervention and documented in the anaesthesia or operative record, including major vascular injury, massive haemorrhage, new-onset arrhythmia or atrial fibrillation, refractory hypertension, or hypercapnic respiratory acidosis.

Estimated blood loss was obtained from anaesthesia follow-up records. Because blood loss was not recorded using a standardised quantitative protocol in routine practice, values of 0 mL indicated the absence of separately documented clinically relevant blood loss rather than complete absence of bleeding. Therefore, this variable was interpreted as documented clinically relevant blood loss rather than a precise quantitative estimate of total intraoperative blood loss, resulting in a markedly zero-inflated distribution.

Vasopressor use was recorded as an intraoperative binary variable. For descriptive clarification, the available anaesthesia and ICU records of patients requiring vasopressor support were re-reviewed to identify the vasopressor agent, mode of administration, whether treatment was transient or remained ongoing at ICU admission, and the available documentation regarding dose and duration. In routine institutional practice, norepinephrine, epinephrine, and dopamine were used for perioperative haemodynamic support. These agents were administered as continuous infusions and titrated according to haemodynamic response within standard adult continuous-infusion dosing ranges reported in pharmacological and critical care references. Norepinephrine is initiated at 0.05–0.1 µg/kg/min and titrated according to haemodynamic response, epinephrine is administered at 0.05–2 µg/kg/min, and dopamine is initiated at 2–5 µg/kg/min with subsequent titration according to clinical response [18]. Because patient-level vasopressor dose trajectories and exact infusion durations were not consistently available across the retrospective records, vasopressor exposure was not analysed quantitatively.

Postoperative variables included the initial postoperative triage pathway, classified as direct ICU admission from the operating room, PACU-to-ICU transfer, or PACU-to-ward transfer. Additional postoperative variables included ICU length of stay, ward length of stay, total hospital length of stay, chest tube duration in days, ICU readmission, atelectasis, pneumonia, postoperative bleeding, postoperative arrhythmia, respiratory failure, reintubation, and 30-day all-cause mortality. For patients transferred from the PACU to the ICU, the documented trigger or triggers for escalation were recorded descriptively when available. ICU readmission was defined as unplanned readmission to the ICU after transfer from the ICU to the surgical ward during the index hospitalisation.

### 2.4. Surgical and Anaesthetic Procedure

All patients received general anaesthesia according to routine institutional thoracic anaesthesia practice. Anaesthetic induction was performed with propofol or thiopental, together with opioid analgesia using fentanyl and neuromuscular blockade with rocuronium. In routine practice, propofol was administered at approximately 1–2 mg/kg or thiopental at approximately 3–5 mg/kg, fentanyl at approximately 1–2 µg/kg as an opioid adjunct, and rocuronium at approximately 0.6 mg/kg to facilitate tracheal intubation. All anaesthetic drug doses were adjusted by the attending anaesthesiologist according to age, body weight, ASA physical status, haemodynamic status, and comorbidity profile. This induction approachis understood to reflect the standing institutional protocol throughout the six-year study period, although clinician-level and temporal variation in agent selection and dosing beyond the parameters described above was not systematically recorded at the patient level and could not be formally assessed.

A double-lumen endotracheal tube was used to facilitate one-lung ventilation. Anaesthesia was maintained with sevoflurane-based balanced general anaesthesia and was adjusted according to clinical response, haemodynamic status, and routine intraoperative monitoring. In routine practice, anaesthetic depth was generally targeted at approximately 0.7–1.3 minimum alveolar concentration (MAC), individualised according to patient age and clinical response, consistent with standard practice to reduce the risk of intraoperative awareness [19]. Target end-tidal sevoflurane concentrations and objective depth-of-anaesthesia monitoring data (e.g., bispectral index or Entropy), postoperative nausea and vomiting risk scores, and detailed information on total intravenous anaesthesia use were not consistently available in the retrospective records and were therefore not included as analytical variables.

During one-lung ventilation, a lung-protective ventilation strategy was applied according to routine institutional thoracic anaesthesia practice, consistent with lung-protective one-lung ventilation approaches described in thoracic anaesthesia literature [20]. Tidal volume was generally set at 4–6 mL/kg predicted body weight. PEEP was commonly initiated at approximately 5 cmH_2_O and subsequently adjusted according to oxygenation, airway pressures, lung compliance, and haemodynamic tolerance. FiO_2_ was titrated to maintain adequate oxygenation and carbon dioxide clearance, with adjustments guided by peripheral oxygen saturation, arterial blood gas results when available, airway pressures, and the intraoperative clinical course. Accordingly, ventilatory settings were individualised within this institutional lung-protective framework rather than applied as fixed settings for all patients. Because granular ventilation trajectories were not consistently available across the six-year study period, ventilation management was described at the protocol level rather than modelled as a candidate predictor.

All procedures were performed by the same thoracic surgery team with curative intent. The surgical approach and extent of resection were individualised according to tumour anatomy, anticipated technical complexity, and the patient’s functional reserve. Minimally invasive approaches, including VATS or RATS, were preferred when technically feasible; open thoracotomy was performed when minimally invasive access was not considered appropriate. Invasive haemodynamic monitoring was applied as clinically indicated, particularly in patients with anticipated haemodynamic instability, substantial cardiopulmonary comorbidity, complex resection, or expected need for postoperative ICU monitoring, and was standard for all patients admitted to the ICU. Therefore, invasive monitoring was not considered a candidate predictor in analyses intended to model actual postoperative ICU admission. Because the indication for invasive monitoring itself reflected clinician risk assessment and anticipated postoperative disposition, its inclusion as a predictor could have introduced additional circularity.

The decision to transfer a patient directly from the operating room to the ICU was made by the responsible anaesthesiologist based on the intraoperative course and early postoperative clinical status. Patients not transferred directly to the ICU were initially observed in the PACU; subsequent transfer to the surgical ward or ICU was determined at reassessment by the same responsible anaesthesiologist. Both direct ICU admission from the operating room and subsequent transfer from the PACU to the ICU were classified as actual postoperative ICU admission. The distinction between these two pathways reflects two clinically different postoperative triage decision points. Direct ICU admission from the operating room represented an immediate end-of-surgery critical care decision made by the responsible anaesthesiologist before the patient left the operating room. In accordance with general perioperative critical care principles and criteria previously used in pulmonary resection studies [12,13], clinical considerations supporting direct ICU admission included inability to extubate safely, ongoing need for invasive mechanical ventilation, inadequate airway protection, severe hypoxaemia or acute respiratory failure, haemodynamic instability or shock requiring vasoactive support, major bleeding or ongoing transfusion requirement, serious cardiac, metabolic, or renal derangements, and major intraoperative clinical or surgical complications requiring ICU-level monitoring.

In contrast, PACU-to-ICU transfer represented escalation after initial post-anaesthesia observation, when clinical deterioration became apparent during early recovery rather than being fully established at operating-room discharge. Because no prespecified institutional protocol or independent adjudication process was used to determine ICU-level care requirements, escalation from the PACU to the ICU was interpreted as observed clinical disposition within routine institutional practice. Relevant triggers included persistent respiratory compromise, oxygen desaturation, hypercapnia or respiratory acidosis, refractory hypertension, haemodynamic instability, new-onset arrhythmia, suspected or confirmed pulmonary embolism, postoperative bleeding, pain-related impairment of respiratory recovery, prolonged PACU stay, or the need for continued high-acuity monitoring. Accordingly, these two pathways were retained as components of actual postoperative ICU admission while being descriptively distinguished because they represent different clinical contexts and decision points. Thus, the primary outcome reflected observed postoperative ICU disposition within routine institutional practice rather than an externally adjudicated ICU-level care requirement.

### 2.5. Development of the Exploratory Perioperative ICU Triage Score

An exploratory perioperative ICU triage score was developed as a descriptive risk stratification approach to translate the primary perioperative model into a simpler point-based format. The score was not intended as a ready-to-implement clinical decision rule, but rather as a hypothesis-generating tool requiring external validation before clinical use. The score components were derived from the clinically pre-specified primary perioperative model, which included operative duration, intraoperative complication, COPD, and pre-existing arrhythmia. Operative duration was dichotomised to improve clinical interpretability, and the optimal threshold was determined using the maximum Youden index derived from ROC curve analysis (Supplementary Table S1). Binary predictors were retained in their original form. Integer point weights were assigned using a pragmatic coefficient-scaled approach anchored to the strongest predictor. Intraoperative complication had the largest effect estimate in the primary model (adjusted OR 15.002; 95% CI, 3.738–60.210; *p* < 0.001; β = 2.708) and was therefore assigned 2 points. For operative duration, the coefficient-scaled raw weight was calculated as (β for operative duration × 180/β for intraoperative complication) × 2 = (0.0115 × 180/2.708) × 2 = 1.529. Despite this raw weight, operative duration was assigned 1 point because it represented a dichotomised continuous exposure rather than an acute adverse event, thereby preserving bedside simplicity and avoiding overweighting a threshold-derived variable. COPD (adjusted OR 1.993; 95% CI, 0.882–4.507; *p* = 0.097; β = 0.690) and pre-existing arrhythmia (adjusted OR 2.338; 95% CI, 0.861–6.351; *p* = 0.096; β = 0.849) were retained as clinically pre-specified baseline cardiopulmonary risk components and were each assigned 1 point. This procedure resulted in 1 point for operative duration ≥ 180 min, 2 points for intraoperative complication, 1 point for COPD, and 1 point for pre-existing arrhythmia. The regression coefficients, scaling procedure, and point assignment rationale for each score component are presented in Supplementary Table S7. A cumulative score was then calculated for each patient by summing the assigned points, yielding a possible score range of 0 to 5.

### 2.6. Statistical Analysis

All analyses were performed using Python (version 3.12.13) via the Google Colaboratory platform (Google LLC, Mountain View, CA, USA). Descriptive data checks and verification of selected outputs were also performed using IBM SPSS Statistics for Windows, version 27.0 (IBM Corp., Armonk, NY, USA). Python code was generated with the assistance of ChatGPT (GPT-5.5 Thinking; OpenAI, San Francisco, CA, USA), with all analytical decisions, variable selections, statistical outputs and interpretations independently reviewed and verified by the authors. The Python libraries used included pandas, NumPy, SciPy, statsmodels, scikit-learn, and matplotlib. The full analytical code is provided as Supplementary File S1. A two-sided *p* value < 0.05 was considered statistically significant. Continuous variables are presented as median and interquartile range [IQR]; categorical variables are presented as number and percentage. Continuous variables were compared between groups using the Mann–Whitney U test for two-group comparisons and the Kruskal–Wallis test for three-group comparisons. Binary categorical variables were compared using Fisher’s exact test when expected cell frequencies were less than five and the continuity-corrected chi-square test otherwise; multilevel categorical variables were compared using Pearson’s chi-square test.

Univariable logistic regression analyses evaluated associations between candidate preoperative and operative variables and actual postoperative ICU admission. The primary multivariable logistic regression model was clinically pre-specified and restricted to four variables—operative duration as a continuous variable, intraoperative complication, COPD, and pre-existing arrhythmia—to limit model complexity relative to the number of outcome events and reduce overfitting risk.

With 45 outcome events and four predictors, the events-per-variable ratio was approximately 11, which informed the decision to avoid data-driven variable selection and adopt clinical pre-specification as the primary modelling strategy. The resulting model was therefore interpreted as a derivation-cohort, hypothesis-generating model requiring external validation before clinical implementation. Although hypertension and estimated blood loss showed statistically significant univariable associations with actual postoperative ICU admission, neither variable was incorporated into the primary model. This decision was made to preserve the pre-specified four-predictor structure and to avoid increasing model complexity relative to the limited number of outcome events. In addition, hypertension was considered susceptible to selection-related confounding in this restricted analytical cohort, whereas estimated blood loss was affected by a markedly zero-inflated documentation pattern. Both variables were therefore evaluated separately in sensitivity analyses, as presented in Supplementary Tables S3 and S4.

A preoperative-only comparator model was constructed to represent information available before surgery and included COPD, pre-existing arrhythmia, FVC%, ASA physical status, and CCI. These variables were selected a priori to reflect respiratory reserve, baseline cardiopulmonary risk, anaesthetic risk classification, and comorbidity burden. The primary perioperative model, the preoperative-only model, and the exploratory perioperative ICU triage score were compared in terms of discrimination and classification performance.

Discriminative performance was assessed using ROC curve analysis and AUC with 95% confidence intervals estimated from 5000 bootstrap resamples. At the Youden-selected optimal cutoff, sensitivity, specificity, positive predictive value (PPV), negative predictive value (NPV), and accuracy were calculated. For the exploratory perioperative ICU triage score, classification metrics were additionally assessed at prespecified score thresholds. Pairwise differences in AUC were evaluated using paired bootstrap resampling with 5000 resamples. Calibration was assessed using a calibration plot, the Brier score, and the Hosmer–Lemeshow goodness-of-fit test. Given that the analytical cohort comprised 159 patients, each decile of predicted probability contained approximately 15–16 observations; calibration estimates should therefore be interpreted as preliminary, as reliable calibration assessment will only be feasible in a larger external validation cohort. Bootstrap internal validation was performed with 1000 resamples. Of these, 983 yielded successful model refits and 17 failed refits, most likely reflecting occasional singularity, complete or near-complete separation, or sparse event patterns in resampled datasets, particularly in relation to the strong association between intraoperative complication and the outcome. The mean optimism estimate (0.015) and optimism-corrected AUC (0.787) were calculated from the 983 successful resamples. Given the low frequency of failed refits (1.7%), the internal validation estimate was considered interpretable, although this instability was acknowledged as a limitation of logistic regression in a modest event-count derivation cohort. Clinical utility was explored using decision curve analysis, comparing net benefit of the three approaches against treat-all and treat-none reference strategies across a range of threshold probabilities. Decision curve findings were considered exploratory and hypothesis-generating within the derivation cohort. No missing data were present for variables included in the primary model, comparator models, or prespecified sensitivity analyses (Supplementary Table S2).

## 3. Results

### 3.1. Study Cohort and Postoperative Triage Flow

**Figure 1:**
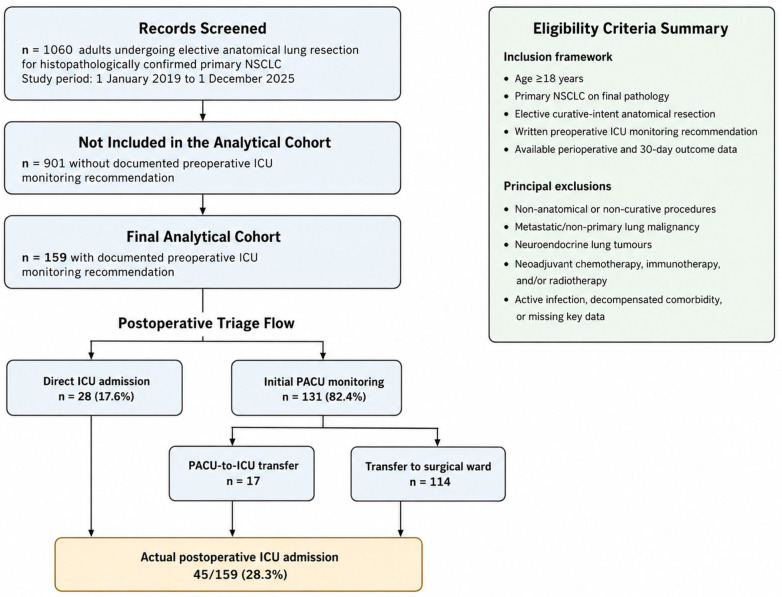
Patient selection and postoperative triage flow. Institutional lung cancer patient records were retrospectively reviewed, and 1060 patients who underwent elective anatomical lung resection for primary non-small cell lung cancer between 1 January 2019, and 1 December 2025, were identified as the screened population for the present analysis. Among these, 159 patients had a documented preoperative recommendation for postoperative ICU monitoring recorded in the routine preoperative anaesthesia assessment and constituted the analytical cohort. All 159 patients fulfilled the prespecified eligibility criteria, none met the predefined exclusion criteria, and no additional patient-level exclusions were applied after cohort identification. The remaining 901 screened patients did not have a documented preoperative ICU monitoring recommendation and were therefore not included in the analytical cohort. Of the 159 included patients, 28 were admitted directly to the ICU from the operating room, whereas 131 were initially monitored in the PACU. Among patients initially monitored in the PACU, 17 were subsequently transferred to the ICU and 114 were transferred to the surgical ward. Overall, actual postoperative ICU admission occurred in 45 of 159 patients (28.3%), comprising both direct ICU admission and subsequent PACU-to-ICU transfer during the index postoperative period. The three postoperative triage pathways were therefore defined as PACU-to-ward transfer/no ICU admission, direct ICU admission, and PACU-to-ICU transfer. ICU, intensive care unit; NSCLC, non-small cell lung cancer; PACU, post-anaesthesia care unit.

Of 1060 screened patients, 901 did not have a documented preoperative ICU monitoring recommendation and were therefore not included in the analytical cohort. The remaining 159 patients had a documented preoperative ICU monitoring recommendation, fulfilled all prespecified eligibility criteria, and none met the predefined exclusion criteria; therefore, no additional patient-level exclusions were applied after cohort identification. The final analytical cohort comprised 159 patients, representing 15.0% of the screened population. Among these, 28 patients (17.6% of the analytical cohort) were admitted directly to the ICU following surgery, whereas 131 (82.4%) were initially monitored in the PACU. Of patients initially observed in the PACU, 17 were subsequently transferred to the ICU and 114 were transferred to the surgical ward. Accordingly, actual postoperative ICU admission occurred in 45 patients (28.3%), including both direct ICU admission from the operating room and subsequent PACU-to-ICU transfer during the index postoperative period. The patient selection process and postoperative triage flow are depicted in Figure 1.

Because direct ICU admission and PACU-to-ICU transfer represent clinically distinct postoperative decision points, baseline and intraoperative characteristics were additionally compared across three triage pathways: no ICU admission, direct ICU admission, and PACU-to-ICU transfer. This descriptive subgroup comparison is provided in Supplementary Table S8. Among the 17 patients transferred from the PACU to the ICU, the primary documented escalation triggers were respiratory acidosis in 5 patients (29.4%), refractory hypertension in 4 (23.5%), new-onset arrhythmia in 3 (17.6%), computed tomography (CT)-confirmed pulmonary embolism in 2 (11.8%), postoperative bleeding in 2 (11.8%), and inadequate pain control in 1 (5.9%); no case had an undocumented trigger. These PACU escalation events were reported descriptively as clinical reasons for subsequent ICU transfer and were not modelled as candidate predictors, because they occurred after the initial postoperative destination decision and were temporally embedded within the outcome pathway.

### 3.2. Baseline and Perioperative Characteristics

Baseline and perioperative characteristics stratified by actual postoperative ICU admission are presented in Table 1. Most demographic, functional, pulmonary, laboratory, and cardiac assessment variables did not differ significantly between groups, with the exception of preoperative SpO_2_ and hypertension. Preoperative SpO_2_ was marginally higher in patients with actual postoperative ICU admission (97.0 [95.0–98.0]% vs. 96.0 [95.0–97.8]%; *p* = 0.049), although the absolute difference was small and the direction of this association is likely attributable to the selected nature of the cohort rather than a biological relationship. Given the selected nature of the analytical cohort and the lack of a plausible biological gradient, this finding was interpreted as a selection-related or clinically negligible association rather than as evidence of a protective or harmful physiological effect. Hypertension was significantly less frequent among patients with ICU admission (26.7% vs. 57.0%; *p* = 0.001). This inverse association was interpreted cautiously and was not considered indicative of a protective biological effect, particularly in view of the selected cohort design and the sensitivity analysis presented in Supplementary Table S3.

COPD (53.3% vs. 36.8%; *p* = 0.085) and pre-existing arrhythmia (24.4% vs. 13.2%; *p* = 0.135) were numerically more frequent among patients with actual ICU admission, although neither difference reached statistical significance. Differences were more pronounced for intraoperative characteristics: patients with ICU admission had a significantly longer operative duration (150.0 [90.0–200.0] min vs. 95.0 [70.0–140.0] min; *p* < 0.001) and a markedly higher frequency of intraoperative complications (26.7% vs. 2.6%; *p* < 0.001). Vasopressor use was observed exclusively in the ICU admission group (13.3% vs. 0.0%; *p* < 0.001). Because vasopressor use perfectly separated the outcome groups, it was described but not entered into regression models. Re-review of the six vasopressor-treated cases showed that vasopressor therapy had been initiated intraoperatively and remained ongoing at the time of ICU admission in all patients. The vasopressor agent continued at ICU admission was norepinephrine in each case. Therefore, none of the vasopressor-treated patients represented a case of brief intraoperative support that had been completely discontinued before leaving the operating room. Although estimated blood loss showed a zero-inflated distribution in which the median value was 0 mL in both groups, the statistically significant difference (*p* < 0.001) reflected the concentration of documented clinically relevant blood loss in the ICU admission group rather than a difference in median blood loss values.

Postoperative outcomes and complications according to ICU admission status are presented in Table 2. Patients with actual ICU admission had significantly longer total hospital length of stay (8.0 [6.0–10.0] days vs. 6.0 [4.0–8.0] days; *p* = 0.001) and chest tube duration (6.0 [3.0–8.0] days vs. 4.0 [2.0–6.0] days; *p* = 0.002). ICU readmission occurred in 13 patients (28.9% of the ICU admission group; 8.2% of the total cohort), all of whom belonged to the actual ICU admission group. This finding was interpreted as a clinically important postoperative course marker rather than as an independent preoperative or intraoperative predictor, because ICU readmission occurred after the initial triage decision. Respiratory failure (22.2% vs. 0.0%; *p* < 0.001), reintubation (13.3% vs. 0.9%; *p* = 0.002), and 30-day mortality (15.6% vs. 0.0%; *p* < 0.001) were significantly more frequent among patients with actual ICU admission.

### 3.3. Factors Associated with Actual Postoperative ICU Admission

#### 3.3.1. Univariable Logistic Regression Analysis

In univariable logistic regression analysis, longer operative duration was associated with increased odds of actual postoperative ICU admission (OR 1.012 per minute; 95% CI, 1.006–1.018; *p* < 0.001). Intraoperative complication showed the strongest univariable association with the outcome (OR 13.455; 95% CI, 3.582–50.543; *p* < 0.001). It should be noted that both operative duration and intraoperative complication may have directly informed the responsible anaesthesiologist’s decision to admit a patient to the ICU; therefore, potential predictor–outcome circularity represents an inherent limitation of the study design and is discussed further in Section 4. Hypertension was inversely associated with actual postoperative ICU admission (OR 0.274; 95% CI, 0.129–0.585; *p* < 0.001); however, this finding was interpreted cautiously because the analytical cohort was restricted to patients with a documented preoperative ICU monitoring recommendation and may therefore be affected by selection-related effects. Estimated blood loss was associated with the outcome in univariable analysis (OR 1.004 per mL; 95% CI, 1.001–1.007; *p* = 0.014). However, because this variable showed a markedly zero-inflated distribution and reflected the absence or presence of separately documented clinically relevant blood loss rather than a standardised quantitative estimate of total blood loss, it was not included in the primary multivariable model. A sensitivity analysis including estimated blood loss is presented in Supplementary Table S4. COPD (OR 1.959; 95% CI, 0.974–3.939; *p* = 0.059) and pre-existing arrhythmia (OR 2.135; 95% CI, 0.894–5.097; *p* = 0.087) showed directionally consistent but non-significant associations with actual postoperative ICU admission. The main univariable results are presented in Table 3, with the complete analysis provided in Supplementary Table S5.

#### 3.3.2. Primary Perioperative Multivariable Model

In the primary multivariable model, operative duration (adjusted OR 1.012 per minute; 95% CI, 1.005–1.018; *p* < 0.001) and intraoperative complication (adjusted OR 15.002; 95% CI, 3.738–60.210; *p* < 0.001) remained significantly associated with actual postoperative ICU admission after adjustment within this selected analytical cohort. COPD (adjusted OR 1.993; 95% CI, 0.882–4.507; *p* = 0.097) and pre-existing arrhythmia (adjusted OR 2.338; 95% CI, 0.861–6.351; *p* = 0.096) showed directionally increased adjusted odds without reaching conventional statistical significance. Given the modest number of outcome events and the derivation-cohort design, these adjusted associations should be interpreted as hypothesis-generating rather than confirmatory. Results are presented in Table 4.

### 3.4. Model Performance, Calibration, Internal Validation, and Clinical Utility

#### 3.4.1. Discrimination and Comparative Performance

**Figure 2:**
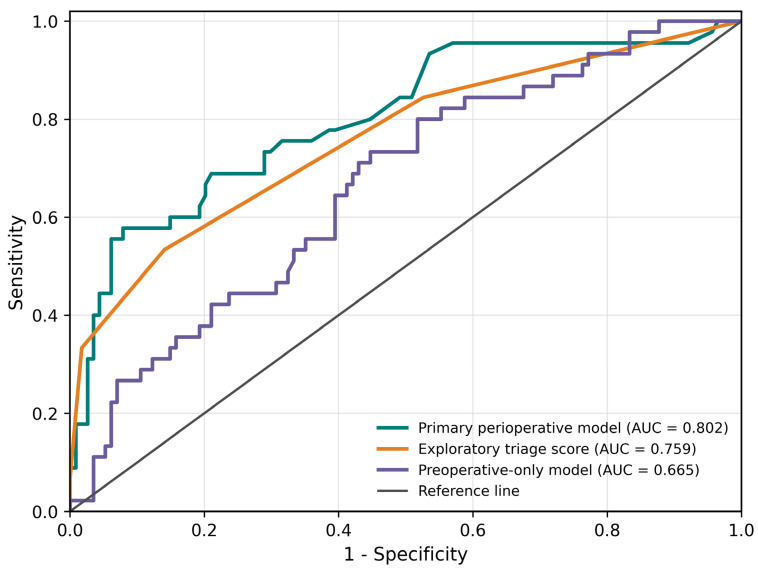
Receiver operating characteristic curves for the three ICU triage approaches. AUC, area under the receiver operating characteristic curve; ICU, intensive care unit.

The primary perioperative multivariable model demonstrated the highest discriminative performance (AUC 0.802; 95% CI, 0.717–0.876). The exploratory perioperative triage score achieved an AUC of 0.759 (95% CI, 0.672–0.839), and the preoperative-only model achieved an AUC of 0.665 (95% CI, 0.573–0.756). At the Youden-selected cutoff of 0.397, the primary model had a sensitivity of 0.578, specificity of 0.921, PPV of 0.743, NPV of 0.847, and accuracy of 0.824. At this threshold, the model showed relatively high specificity but modest sensitivity, indicating a more conservative classification profile for identifying observed ICU admission rather than a maximally sensitive approach for detecting all patients who experienced actual postoperative ICU admission. Given that the outcome prevalence in this pre-selected cohort was only 28.3%, this specificity estimate should not be interpreted as definitive evidence that the model would safely minimise unnecessary ICU admissions in clinical practice. Rather, it should be interpreted in the context of the selected cohort and imbalanced outcome distribution. In pairwise bootstrap comparisons, the primary model significantly outperformed the preoperative-only model (AUC difference, 0.137; 95% CI, 0.038–0.234; *p* = 0.006), and the exploratory score also outperformed the preoperative-only model (AUC difference, 0.094; 95% CI, 0.007–0.181; *p* = 0.033). The difference between the primary model and the exploratory score did not reach statistical significance (AUC difference, 0.042; 95% CI, −0.0004 to 0.090; *p* = 0.053). Discriminative and classification performance are summarised in Table 5A,B, and ROC curves are shown in Figure 2.

#### 3.4.2. Calibration and Internal Validation

**Figure 3:**
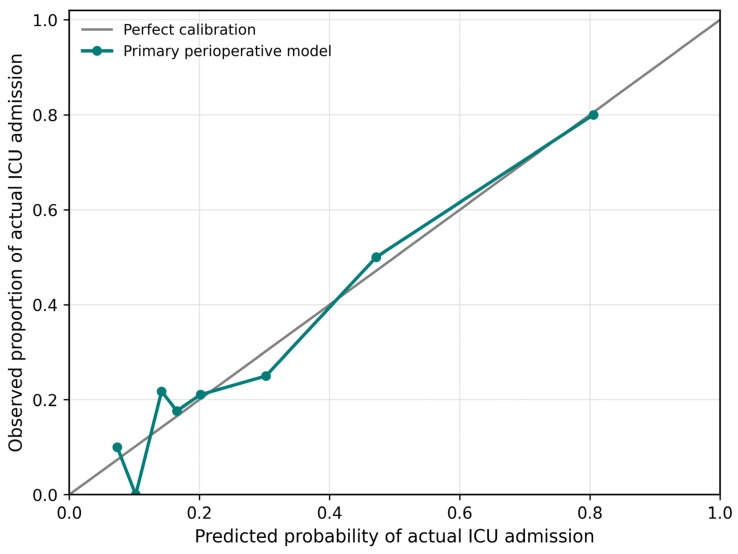
Calibration plot for the primary perioperative multivariable model. Each point represents a decile of predicted probability plotted against the observed proportion of actual postoperative ICU admission. The diagonal line indicates perfect calibration. Because each decile included a limited number of patients, the plot should be interpreted as a preliminary assessment of calibration. ICU, intensive care unit.

The calibration plot suggested preliminary visual agreement between predicted probabilities and observed ICU admission proportions (Figure 3). The Brier score was 0.147, and the Hosmer–Lemeshow test did not indicate statistically significant miscalibration (χ^2^ = 12.659, df = 8; *p* = 0.124). However, because the analytical cohort included only 159 patients and calibration was assessed across deciles of predicted probability, each calibration bin contained approximately 15–16 observations. Therefore, the calibration plot and Hosmer–Lemeshow test should be interpreted cautiously and considered preliminary descriptive assessments rather than definitive evidence of model calibration. Of 1000 requested bootstrap resamples, 983 yielded successful model refits; 17 resamples failed, most likely because of occasional complete or near-complete separation or sparse event patterns in resampled datasets. The mean optimism was 0.015, and the optimism-corrected AUC was 0.787. Because failed refits represented only a small proportion of the requested resamples, the internal validation estimate was considered interpretable, although it remains exploratory and requires confirmation in an external validation cohort. Calibration and internal validation metrics are provided in Supplementary Table S6.

#### 3.4.3. Clinical Utility

**Figure 4:**
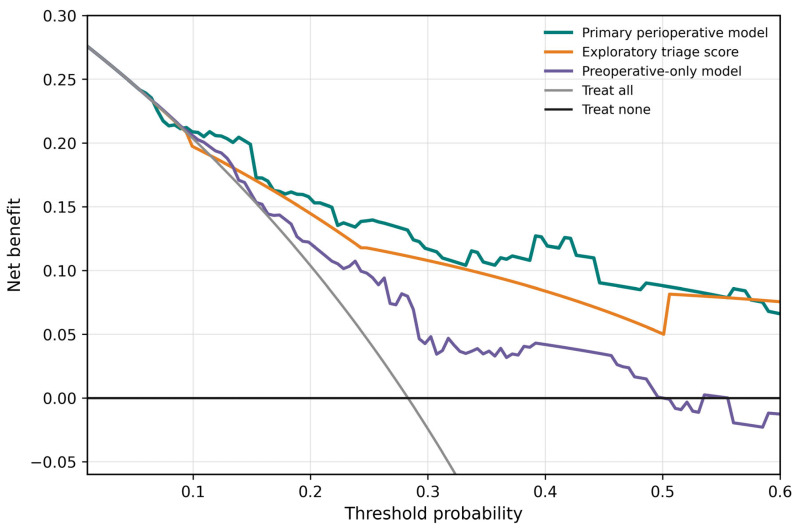
Decision curve analysis for the primary perioperative multivariable model, the exploratory perioperative ICU triage score, and the preoperative-only model. The analysis compares the net benefit of each approach against treat-all and treat-none reference strategies across threshold probabilities. Decision curve findings were derived from the same cohort used for model development and should therefore be interpreted as exploratory. ICU, intensive care unit.

Decision curve analysis (Figure 4) demonstrated that the primary perioperative multivariable model provided greater net benefit than the preoperative-only model over most of the examined threshold-probability range and showed higher net benefit than the treat-all and treat-none reference strategies across clinically relevant portions of this range. Net benefit from the primary model was broadly comparable to, and over parts of the range numerically greater than, that from the exploratory triage score. Because all models were developed and evaluated in the same single-centre cohort, these findings should be interpreted as exploratory and hypothesis-generating rather than as evidence supporting immediate clinical implementation.

### 3.5. Exploratory Perioperative ICU Triage Score

**Figure 5:**
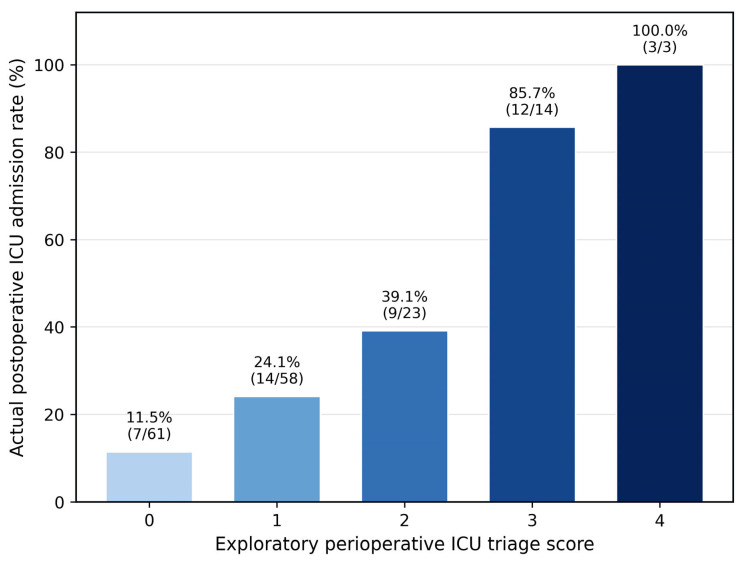
Actual postoperative ICU admission rates by exploratory perioperative ICU triage score category. Numbers above the bars indicate the proportion (%) and count (*n*/total) of patients with actual postoperative ICU admission within each score category. The score 4 category included only three patients; therefore, this estimate should be interpreted descriptively. ICU, intensive care unit.

The exploratory perioperative triage score demonstrated a progressive increase in observed actual postoperative ICU admission rates across score categories: 11.5% (7/61) at score 0, 24.1% (14/58) at score 1, 39.1% (9/23) at score 2, 85.7% (12/14) at score 3, and 100.0% (3/3) at score 4 (Table 6A,B; Figure 5). No patient had a score of 5. Because the score 4 category included only three patients, the 100.0% admission rate should be interpreted as a descriptive finding rather than a stable risk estimate. A threshold of ≥1 point offered high sensitivity (0.844) with limited specificity (0.474); a threshold of ≥2 points provided a more balanced profile (sensitivity 0.533, specificity 0.860, accuracy 0.767); and thresholds of ≥3 and ≥4 points yielded high specificity and PPV at the cost of markedly reduced sensitivity. Because the score and its operative-duration threshold were derived and evaluated within the same cohort, these findings should be interpreted as hypothesis-generating and should not be considered ready for clinical implementation without external validation.

## 4. Discussion

Among patients with a documented preoperative recommendation for postoperative ICU monitoring following anatomical lung resection for NSCLC, actual postoperative ICU admission occurred in fewer than one-third of patients (45/159, 28.3%). Because the outcome reflected observed postoperative ICU disposition rather than independently adjudicated ICU-level care requirement, these findings should be interpreted within the institutional context in which triage decisions were made. The principal finding of this study is that perioperative information available at the conclusion of surgery—particularly operative duration and documented intraoperative complication—was more strongly associated with actual postoperative ICU admission than the evaluated preoperative-only approach. The primary perioperative multivariable model achieved an AUC of 0.802, and the exploratory perioperative triage score demonstrated a clinically interpretable risk gradient with an AUC of 0.759. However, both the model and the score were developed and evaluated in the same single-centre derivation cohort, and their findings should therefore be regarded as hypothesis-generating. Taken together, these findings suggest that a documented preoperative ICU monitoring recommendation may be better conceptualised as an initial planning framework subject to structured perioperative reassessment rather than as a definitive postoperative disposition decision.

### 4.1. Discordance Between Documented Preoperative Recommendation and Actual ICU Admission

The finding that actual postoperative ICU admission occurred in only 28.3% of patients with a documented preoperative recommendation for ICU monitoring highlights the distinction between anticipatory planning and observed clinical disposition. The present study evaluated actual ICU admission rather than an externally adjudicated or standardised definition of mandatory ICU requirement; accordingly, the observed proportion does not indicate that preoperative recommendations were inappropriate, but rather that the eventual care pathway in this selected cohort was substantially shaped by information that emerged during and immediately after surgery. In addition, actual ICU admission may have been influenced by institutional resources, PACU capacity, ICU bed availability, and clinician-level decision thresholds. Therefore, the primary outcome should be interpreted as observed disposition behaviour rather than as a direct surrogate for independently adjudicated ICU-level care requirement. Consequently, the absence of predefined and externally adjudicated ICU admission criteria may have reduced the internal validity of the observed associations and limits the generalisability of the findings to institutions with different postoperative triage practices and resource availability.

The pooled primary outcome also combined two clinically distinct postoperative pathways: direct ICU admission from the operating room and subsequent PACU-to-ICU transfer. These pathways represent different decision points, with potentially different timing, clinical context, and triggers for escalation. In the present study, they were pooled because both represented escalation to ICU-level postoperative monitoring during the index postoperative period; nevertheless, this heterogeneity should be recognised when interpreting the model. For this reason, the two pathways were explicitly defined in the Methods section, descriptively separated in the postoperative triage flow, and compared across three triage groups in Supplementary Table S8. Pathway-specific regression was not pursued because the direct ICU admission and PACU-to-ICU subgroups were small, and such analyses would have been statistically unstable. The descriptive comparison of the three triage pathways and the available documented PACU-to-ICU escalation triggers were therefore summarised to provide additional clinical context for this pooled outcome.This interpretation is consistent with previously published evidence, despite differences in outcome definitions and cohort selection. Pinheiro and colleagues, in a prospective cohort of 120 patients undergoing elective pulmonary resection, reported a sensitivity of 53.3% and specificity of 91.0% for preoperative criteria-based prediction of ICU admission, indicating that preoperative assessment alone may incompletely capture those who ultimately meet predefined ICU criteria [13]. Among 340 patients for whom preoperative ICU reservation had been requested before major lung resection, Kim and colleagues found that only a subset ultimately met criteria for mandatory postoperative critical care; on multivariable analysis, greater intraoperative haemorrhage and open thoracotomy were independently associated with this outcome [12]. Although these studies did not employ identical outcome definitions, their findings collectively support integrating perioperative information into postoperative critical care planning. The present study should not be interpreted as suggesting that preoperative evaluation should be abandoned; rather, it indicates that, in patients already flagged for potential ICU monitoring, a final disposition decision informed by the intraoperative course may better reflect the patient’s immediate postoperative trajectory within a dynamic, institution-specific triage pathway.

### 4.2. Perioperative Factors and the Contribution of Intraoperative Information

Operative duration and intraoperative complication retained significant independent associations with actual postoperative ICU admission after adjustment in the primary model. Each additional minute of operative duration was associated with increased odds of ICU admission (adjusted OR 1.012; 95% CI, 1.005–1.018), and a documented intraoperative complication was associated with markedly elevated odds (adjusted OR 15.002; 95% CI, 3.738–60.210). These associations are clinically plausible: prolonged operative duration may reflect greater technical complexity and cumulative anaesthetic and physiological burden, while clinically significant intraoperative adverse events may directly prompt escalation of postoperative care.

These findings should be interpreted as associations rather than causal relationships. Because operative duration and intraoperative complication were also available to the responsible anaesthesiologist at the time of postoperative disposition, they may have directly informed the ICU admission decision. This creates potential predictor–outcome circularity, particularly because the outcome was actual postoperative ICU admission rather than externally adjudicated ICU requirement. Therefore, the primary model should be viewed as characterising observed triage behaviour in this institutional setting rather than as identifying causal determinants of independently adjudicated ICU-level care requirement. The Surgical Apgar Score and subsequent validation studies established the broader concept that intraoperative physiological and procedural information can meaningfully contribute to short-term postoperative risk stratification [21,22]. The present study extends this concept to a focused cohort in whom ICU monitoring had already been recommended preoperatively, and identifies a practical role for end-of-surgery reassessment using variables available before the patient leaves the operative environment. COPD and pre-existing arrhythmia were retained in the primary model as clinically relevant cardiopulmonary risk domains; both were associated with directionally increased adjusted odds of ICU admission, although neither reached conventional statistical significance in this cohort, consistent with their borderline univariable associations.

Vasopressor requirement was observed exclusively among patients who experienced actual postoperative ICU admission and was therefore not entered into logistic regression because of complete separation. Clinically, this finding supports the relevance of intraoperative haemodynamic deterioration to postoperative disposition. However, vasopressor use should be interpreted descriptively, as the distinction between transient intraoperative support and ongoing vasopressor dependence at ICU admission is important for understanding whether vasopressor treatment reflected a reversible intraoperative event or persistent postoperative instability. In the present cohort, re-review of the six vasopressor-treated patients showed that all were admitted to the ICU with ongoing norepinephrine infusion initiated intraoperatively. This finding suggests that vasopressor requirement in this subgroup did not merely reflect transient, reversible intraoperative hypotension, but rather persistent perioperative haemodynamic instability at the time of postoperative disposition. Accordingly, vasopressor use was reported descriptively and interpreted as a clinically relevant marker that may have directly informed the ICU admission decision.

Hypertension was inversely associated with actual postoperative ICU admission in univariable analysis. Given that the cohort was restricted to patients with a documented preoperative ICU monitoring recommendation, this unexpected direction of association may reflect selection processes, clinical decision-making patterns, or residual confounding, and is not interpreted as evidence of a biological protective effect [23]. One possible explanation is that hypertension in this selected cohort may have represented a well-characterised and medically optimised comorbidity profile, whereas the absence of hypertension may have identified patients selected for ICU monitoring because of other less predictable perioperative risks. This interpretation is supported by the sensitivity analysis including hypertension, in which the adjusted odds ratio for hypertension was 0.422 (95% CI, 0.177–1.002; *p* = 0.051), attenuated compared with the univariable estimate and not achieving conventional statistical significance, while the primary model predictors remained robust (Supplementary Table S3).

Preoperative SpO_2_ was marginally higher among patients who experienced actual postoperative ICU admission, an apparently paradoxical finding. This should not be interpreted as indicating that higher oxygen saturation increased postoperative ICU admission risk. Rather, within a cohort already selected for preoperative ICU monitoring recommendation, patients with lower baseline SpO_2_ may have undergone closer preoperative optimisation, been selected differently for surgery, or been managed with alternative perioperative strategies. This selection effect may have obscured expected physiological gradients for baseline respiratory variables.

ICU readmission after initial ICU-to-ward transfer occurred in 13 patients and was observed only among those who had experienced actual ICU admission. Although ICU readmission occurred after the initial triage decision and was therefore not considered a candidate predictor, it is clinically relevant. It may reflect postoperative deterioration despite initial ICU monitoring, premature step-down, or progression of complications not fully predictable at the time of initial disposition. Accordingly, ICU readmission should be interpreted as an important postoperative course marker and as a reminder that initial ICU triage and subsequent step-down decisions represent related but distinct components of perioperative critical care planning.

### 4.3. Exploratory Perioperative ICU Triage Score and Predictive Performance

The exploratory score demonstrated a monotonically increasing gradient in observed ICU admission rates across score categories (11.5% to 100.0%), consistent with a clinically interpretable observed risk gradient. However, the highest score category included only three patients; therefore, the 100.0% observed admission rate should be interpreted descriptively rather than as a stable risk estimate. At a threshold of ≥2 points, the score offered specificity of 0.860 and NPV of 0.824; at ≥3 points, PPV increased to 0.882 at the cost of substantially reduced sensitivity. The comparative analysis showed that both the primary perioperative model and the exploratory score significantly outperformed the preoperative-only model, whereas the numerical difference in AUC between the primary model (0.802) and the exploratory score (0.759) did not reach statistical significance (*p* = 0.053). This finding should not be interpreted as evidence of equivalence, but rather as suggesting that a simpler perioperative point-based approach may preserve clinically relevant discriminative performance and warrants prospective evaluation.

The primary model demonstrated generally acceptable apparent calibration (Brier score 0.147; Hosmer–Lemeshow *p* = 0.124), with an optimism-corrected AUC of 0.787 following bootstrap internal validation. These calibration results should be interpreted cautiously because the cohort included only 159 patients, and decile-based calibration assessment resulted in approximately 15–16 observations per calibration bin. Therefore, the calibration plot and Hosmer–Lemeshow test provide only a preliminary assessment of calibration. Decision curve analysis provided exploratory evidence of net benefit for the primary model over reference strategies across a clinically relevant range of threshold probabilities. These findings are encouraging but do not establish clinical utility outside the derivation setting. Similarly, the small number of failed bootstrap refits likely reflected sparse event patterns or complete/near-complete separation in some resampled datasets, reinforcing that the internal validation findings should be considered exploratory pending external validation.

### 4.4. Comparison with Existing Evidence

Established thoracic surgical risk assessment tools remain valuable for preoperative counselling and operative selection but are not designed to determine ICU disposition after the intraoperative course is known. Related work has similarly questioned the adequacy of preoperative-only ICU planning before elective surgery [24]. Wang and colleagues, evaluating 1321 patients with NSCLC, developed a model for postoperative unplanned ICU admission incorporating the modified systemic inflammation score, alcohol use, intraoperative fluid volume, and preoperative comorbidities, reporting a C-statistic of 0.799 [25]. Although their outcome and cohort differed, their findings similarly indicate that intraoperative information adds value to ICU-related risk assessment after lung cancer surgery. More recently, Özçıbık Işık and colleagues applied deep learning models to 953 patients undergoing NSCLC surgery and reported a test AUC of 0.83 for postoperative ICU admission [26]. The comparable range of discriminative performance across these diverse methodological approaches suggests that a clinically transparent perioperative point-based score may offer potential advantages in terms of bedside interpretability, provided that it undergoes external validation in prospective multicentre settings.

### 4.5. Strengths and Limitations

A principal strength of this study is its well-defined analytical cohort: all included patients had a documented preoperative recommendation for ICU monitoring recorded during routine anaesthetic assessment. This design allowed examination of a pragmatic and resource-relevant clinical question within a clearly identified high-risk subgroup. The analysis clearly distinguished a primary perioperative regression model from an explicitly exploratory score, evaluated discrimination, calibration, and internal validity, and explored potential clinical utility through decision curve analysis.

Several limitations require consideration. First, this was a retrospective, single-centre study. ICU admission decisions may have been influenced by institutional resource availability, local perioperative pathways, and individual clinician judgement in addition to patient condition. The outcome was actual postoperative ICU admission in a real-world setting rather than a standardised, externally adjudicated definition of ICU requirement; consequently, the model characterises observed disposition in this institutional context. This limits both internal validity and generalisability, particularly because practice patterns, ICU bed availability, PACU capacity, and clinician thresholds may vary across centres and over time. Second, the analytical cohort comprised patients with a documented preoperative ICU monitoring recommendation only (15.0% of the screened population), which appropriately focuses the analysis but limits generalisability to all patients undergoing anatomical lung resection. Detailed baseline characteristics of the 901 patients without documented preoperative ICU monitoring recommendation were not available for comparative analysis, and criterion-specific exclusion counts beyond the absence of preoperative ICU recommendation could not be reliably reconstructed from the available retrospective dataset. Therefore, residual selection bias cannot be excluded. Third, the number of outcome events was modest (*n* = 45), restricting model complexity and the reliability of subgroup analyses; in particular, the exploratory score and its operative-duration threshold were derived and evaluated within the same cohort, and their reported performance should be regarded as hypothesis-generating. Although clinical pre-specification was used to avoid data-driven variable selection, the model remains a derivation-cohort model requiring external validation in an adequately powerd cohort before clinical use. Fourth, estimated blood loss was affected by a zero-inflated documentation pattern in which values of 0 mL reflected the absence of separately documented clinically relevant bleeding; this precluded its inclusion in the primary model. Fifth, detailed intraoperative physiological variables—including haemodynamic trajectories, cumulative fluid balance, transfusion requirements, and ventilatory parameters—were not uniformly available for modelling. Similarly, anaesthetic depth monitoring, detailed one-lung ventilation parameters, PONV risk assessment, TIVA use, and precise criteria for invasive haemodynamic monitoring were not consistently available across the six-year period. These unmeasured perioperative management factors may have contributed to intraoperative complications, postoperative respiratory outcomes, and clinician disposition decisions.

Finally, because direct ICU admission and PACU-to-ICU transfer were pooled in the primary outcome, pathway-specific predictors could not be robustly modelled separately because of limited subgroup sample sizes. The descriptive subgroup comparison and available PACU-to-ICU trigger analysis were therefore intended to contextualise, rather than fully resolve, the heterogeneity of the pooled outcome.

### 4.6. Clinical Implications

These findings support further evaluation of a two-stage approach to postoperative ICU disposition assessment in patients undergoing anatomical lung resection for NSCLC. A preoperative recommendation for ICU monitoring remains valuable for anticipating care needs and preparing resources. However, the final disposition decision may benefit from structured reassessment at the end of surgery or during early PACU observation, incorporating the intraoperative course alongside relevant preoperative cardiopulmonary risk factors. The exploratory score provides a transparent framework for such reassessment but should not yet be applied as a stand-alone decision tool. Prospective multicentre validation with predefined ICU admission criteria and capture of granular intraoperative physiological variables is required to determine whether perioperative reassessment can improve triage consistency, clinical decision-making, and resource allocation beyond current clinical judgement. Until such validation is available, the present findings should be interpreted as supporting further evaluation of structured perioperative reassessment rather than immediate implementation of the proposed model or score in routine clinical practice.

## 5. Conclusions

In this cohort of patients undergoing anatomical lung resection for NSCLC with a documented preoperative recommendation for postoperative ICU monitoring, actual postoperative ICU admission occurred in 28.3% of patients. Longer operative duration and the presence of an intraoperative complication remained significantly associated with actual postoperative ICU admission after adjustment for COPD and pre-existing arrhythmia. Because the outcome represented observed postoperative ICU disposition rather than independently adjudicated ICU-level care requirement, these findings should be interpreted within the context of this selected single-centre cohort. Perioperative approaches incorporating intraoperative information demonstrated higher apparent discriminative performance than the preoperative-only model. The exploratory perioperative ICU triage score showed a clinically interpretable observed gradient in ICU admission rates across score categories and may provide a transparent, hypothesis-generating framework for perioperative reassessment at the end of surgery. However, the primary model and exploratory score were derived and internally validated within a single retrospective cohort. External validation in prospective, multicentre settings using predefined ICU admission criteria is required before either approach can be considered for routine clinical implementation in postoperative ICU triage following thoracic oncological surgery.

## Figures and Tables

**Table 1:** Baseline and operative characteristics according to actual postoperative ICU admission.

Variable	Overall (*n* = 159)	No ICU Admission (*n* = 114)	ICU Admission (*n* = 45)	*p* Value
Preoperative Characteristics				
Age, years	66.0 [60.0–72.0]	66.0 [60.2–71.0]	66.0 [60.0–72.0]	0.780
Body mass index, kg/m^2^	26.1 [23.7–30.0]	26.4 [23.7–30.1]	25.9 [23.7–28.4]	0.528
Smoking exposure, pack-years	40.0 [0.0–51.5]	40.0 [0.0–52.2]	35.0 [15.0–50.0]	0.882
FEV_1_, % predicted	78.0 [61.0–95.0]	79.5 [60.2–96.0]	75.0 [63.0–92.0]	0.595
FVC, % predicted	76.0 [65.0–90.0]	78.5 [65.5–91.0]	73.0 [64.0–87.0]	0.212
FEV_1_/FVC, % predicted	107.0 [97.0–114.5]	106.0 [95.0–114.0]	108.0 [98.0–115.0]	0.419
ASA physical status	3.0 [2.0–3.0]	3.0 [3.0–3.0]	3.0 [2.0–3.0]	0.111
ECOG performance status	2.0 [1.0–2.0]	2.0 [1.0–2.0]	1.0 [1.0–2.0]	0.365
Charlson Comorbidity Index	6.0 [5.0–8.0]	7.0 [5.0–8.0]	6.0 [5.0–8.0]	0.457
Modified Frailty Index-11	0.2 [0.1–0.3]	0.2 [0.1–0.3]	0.2 [0.1–0.3]	0.983
Preoperative SpO_2_, %	97.0 [95.0–98.0]	96.0 [95.0–97.8]	97.0 [95.0–98.0]	0.049
C-reactive protein, mg/L	4.0 [3.0–5.0]	4.0 [3.0–5.0]	4.0 [3.0–5.0]	0.468
Male sex	117 (73.6)	80 (70.2)	37 (82.2)	0.176
Hypertension	77 (48.4)	65 (57.0)	12 (26.7)	0.001
Diabetes mellitus	46 (28.9)	35 (30.7)	11 (24.4)	0.555
COPD	66 (41.5)	42 (36.8)	24 (53.3)	0.085
Coronary artery disease	65 (40.9)	44 (38.6)	21 (46.7)	0.451
Pre-existing arrhythmia	26 (16.4)	15 (13.2)	11 (24.4)	0.135
Valvular heart disease	32 (20.1)	21 (18.4)	11 (24.4)	0.526
Previous coronary angioplasty/stent	56 (35.2)	37 (32.5)	19 (42.2)	0.329
Previous cerebrovascular event	7 (4.4)	5 (4.4)	2 (4.4)	1.000
Heart failure	19 (11.9)	15 (13.2)	4 (8.9)	0.634
Echocardiographic assessment				0.963
Normal	16 (10.1)	11 (9.6)	5 (11.1)	
Pathological	50 (31.4)	36 (31.6)	14 (31.1)	
Not clinically indicated †	93 (58.5)	67 (58.8)	26 (57.8)	
Operative Characteristics				
Surgical approach				0.552
VATS	72 (45.3)	54 (47.4)	18 (40.0)	
Robotic-assisted (RATS)	1 (0.6)	1 (0.9)	0 (0.0)	
Open thoracotomy	86 (54.1)	59 (51.8)	27 (60.0)	
Resection type				0.380
Segmentectomy	58 (36.5)	45 (39.5)	13 (28.9)	
Lobectomy	94 (59.1)	65 (57.0)	29 (64.4)	
Bilobectomy	1 (0.6)	1 (0.9)	0 (0.0)	
Pneumonectomy	6 (3.8)	3 (2.6)	3 (6.7)	
Operative duration, min	100.0 [70.0–165.0]	95.0 [70.0–140.0]	150.0 [90.0–200.0]	<0.001
Estimated blood loss, mL ‡	0.0 [0.0–0.0]	0.0 [0.0–0.0]	0.0 [0.0–0.0]	<0.001
Intraoperative complication	15 (9.4)	3 (2.6)	12 (26.7)	<0.001
Vasopressor requirement §	6 (3.8)	0 (0.0)	6 (13.3)	<0.001

Note. Data are presented as median [interquartile range] or number (%), as appropriate. *p* values were calculated using the Mann–Whitney U test for continuous variables. Binary categorical variables were compared using Fisher’s exact test when expected cell frequencies were less than five and the continuity-corrected chi-square test otherwise; multilevel categorical variables were compared using Pearson’s chi-square test. † Echocardiography was not requested following cardiovascular evaluation; this category does not represent missing data. ‡ Values recorded as 0 mL indicated the absence of separately documented clinically relevant blood loss rather than complete absence of bleeding; this variable showed a markedly zero-inflated distribution. The statistically significant group difference should therefore be interpreted as reflecting documented clinically relevant blood loss rather than a difference in median blood loss values. § Vasopressor use reflected intraoperative haemodynamic deterioration; because all patients receiving vasopressors experienced actual ICU admission, this variable was not entered into regression models owing to complete separation. Re-review of these six patients showed that all were transferred to the ICU with ongoing norepinephrine infusion initiated intraoperatively; therefore, vasopressor support was interpreted as persistent perioperative haemodynamic instability rather than transient intraoperative support alone. ASA, American Society of Anesthesiologists; CCI, Charlson Comorbidity Index; COPD, chronic obstructive pulmonary disease; ECOG, Eastern Cooperative Oncology Group; FEV_1_, forced expiratory volume in one second; FEV_1_/FVC, forced expiratory volume in one second to forced vital capacity ratio, expressed as a percentage; FVC, forced vital capacity; ICU, intensive care unit; IQR, interquartile range; RATS, robot-assisted thoracoscopic surgery; SpO_2_, peripheral oxygen saturation; VATS, video-assisted thoracoscopic surgery.

**Table 2:** Postoperative outcomes and complications according to actual postoperative ICU admission.

Variable	Overall (*n* = 159)	No ICU Admission (*n* = 114)	ICU Admission (*n* = 45)	*p* Value
Initial postoperative unit				<0.001
PACU	131 (82.4)	114 (100.0)	17 (37.8)	
Direct ICU admission	28 (17.6)	0 (0.0)	28 (62.2)	
ICU length of stay, days	0.0 [0.0–1.0]	0.0 [0.0–0.0]	2.0 [1.0–5.0]	<0.001
Ward length of stay, days	6.0 [3.0–8.0]	6.0 [4.0–8.0]	5.0 [2.0–7.0]	0.126
Total hospital stay, days	6.0 [4.0–9.0]	6.0 [4.0–8.0]	8.0 [6.0–10.0]	0.001
Chest tube duration, days	4.0 [3.0–6.0]	4.0 [2.0–6.0]	6.0 [3.0–8.0]	0.002
ICU readmission	13 (8.2)	0 (0.0)	13 (28.9)	<0.001
Atelectasis	9 (5.7)	1 (0.9)	8 (17.8)	<0.001
Pneumonia	5 (3.1)	1 (0.9)	4 (8.9)	0.023
Postoperative bleeding	2 (1.3)	0 (0.0)	2 (4.4)	0.079
Postoperative arrhythmia	3 (1.9)	0 (0.0)	3 (6.7)	0.022
Respiratory failure	10 (6.3)	0 (0.0)	10 (22.2)	<0.001
Reintubation	7 (4.4)	1 (0.9)	6 (13.3)	0.002
30-day all-cause mortality	7 (4.4)	0 (0.0)	7 (15.6)	<0.001

Note. Data are presented as median [interquartile range] or number (%), as appropriate. *p* values were calculated using the Mann–Whitney U test for continuous variables and Fisher’s exact test or the continuity-corrected chi-square test for categorical variables, as appropriate. ICU, intensive care unit; PACU, post-anaesthesia care unit.

**Table 3:** Univariable logistic regression analysis for actual postoperative ICU admission.

Variable	Unadjusted OR (95% CI)	*p* Value
Patient Characteristics		
Age, per year	1.002 (0.968–1.038)	0.902
Body mass index, per kg/m^2^	0.976 (0.919–1.036)	0.420
Smoking exposure, per pack-year	1.001 (0.991–1.012)	0.811
Male sex	1.966 (0.829–4.660)	0.125
Pulmonary and Laboratory Variables		
FEV_1_, per % predicted	0.996 (0.981–1.011)	0.612
FVC, per % predicted	0.988 (0.969–1.007)	0.226
FEV_1_/FVC, per %	1.011 (0.988–1.034)	0.355
Preoperative SpO_2_, per %	1.114 (0.953–1.301)	0.176
C-reactive protein, per mg/L	1.079 (0.909–1.282)	0.383
Clinical Status		
ASA physical status, per class	0.651 (0.330–1.282)	0.214
ECOG performance status, per point	0.888 (0.540–1.461)	0.640
Charlson Comorbidity Index, per point	0.929 (0.799–1.081)	0.342
Modified Frailty Index-11, per point	0.959 (0.044–21.043)	0.979
Comorbidities		
Hypertension	0.274 (0.128–0.585)	<0.001
Diabetes mellitus	0.730 (0.332–1.605)	0.434
COPD	1.959 (0.974–3.939)	0.059
Coronary artery disease	1.392 (0.694–2.794)	0.352
Pre-existing arrhythmia	2.135 (0.894–5.097)	0.087
Valvular heart disease	1.433 (0.626–3.281)	0.395
Previous coronary angioplasty/stent	1.521 (0.748–3.092)	0.247
Previous cerebrovascular event	1.014 (0.189–5.426)	0.987
Heart failure	0.644 (0.202–2.057)	0.458
Operative Factors		
Operative duration, per minute	1.012 (1.006–1.018)	<0.001
Estimated blood loss, per mL ‡	1.004 (1.001–1.007)	0.014
Intraoperative complication	13.455 (3.582–50.543)	<0.001

Note. Odds ratios represent univariable associations with actual postoperative ICU admission. Continuous variables were analysed in their original scale. Variables affected by complete or quasi-complete separation, including vasopressor requirement, were not entered into logistic regression and were described separately. ‡ Estimated blood loss showed a markedly zero-inflated distribution; a recorded value of 0 mL indicated the absence of separately documented clinically relevant blood loss rather than complete absence of bleeding. ASA, American Society of Anesthesiologists; CCI, Charlson Comorbidity Index; CI, confidence interval; COPD, chronic obstructive pulmonary disease; ECOG, Eastern Cooperative Oncology Group; FEV_1_, forced expiratory volume in one second; FEV_1_/FVC, forced expiratory volume in one second to forced vital capacity ratio, expressed as a percentage; FVC, forced vital capacity; ICU, intensive care unit; OR, odds ratio; SpO_2_, peripheral oxygen saturation.

**Table 4:** Primary perioperative multivariable logistic regression model for actual postoperative ICU admission.

Variable	Adjusted OR (95% CI)	*p* Value
Operative duration, per minute	1.012 (1.005–1.018)	<0.001
Intraoperative complication	15.002 (3.738–60.210)	<0.001
COPD	1.993 (0.882–4.507)	0.097
Pre-existing arrhythmia	2.338 (0.861–6.351)	0.096

Note. Adjusted odds ratios were estimated using multivariable logistic regression. The clinically pre-specified primary model included operative duration as a continuous variable, intraoperative complication, COPD, and pre-existing arrhythmia. The model was developed in a single-centre derivation cohort and should be considered exploratory pending external validation. CI, confidence interval; COPD, chronic obstructive pulmonary disease; ICU, intensive care unit; OR, odds ratio.

**Table 5:** (**A**). Discriminative and classification performance of ICU triage approaches. (**B**). Pairwise bootstrap comparison of AUC values.

(**A**)							
**Approach**	**AUC (95% CI)**	**Cutoff**	**Sn**	**Sp**	**PPV**	**NPV**	**Accuracy**
Preoperative-only model	0.665 (0.573–0.756)	0.279	0.733	0.553	0.393	0.840	0.604
Exploratory perioperative triage score	0.759 (0.672–0.839)	≥2	0.533	0.860	0.600	0.824	0.767
Primary perioperative model	0.802 (0.717–0.876)	0.397	0.578	0.921	0.743	0.847	0.824
(**B**)							
**Comparison**		**AUC Difference**		**95% CI for** **Difference**		***p* Value**	
Primary model vs. preoperative-only model		0.137		0.038–0.234		0.006	
Primary model vs. exploratory score		0.042		−0.0004 to 0.090		0.053	
Exploratory score vs. preoperative-only model		0.094		0.007–0.181		0.033	

(**A**) Note. AUC confidence intervals were estimated using bootstrap resampling (5000 resamples). Optimal cutoffs for the model-based predicted probabilities were selected using the Youden index. For the exploratory perioperative triage score, classification metrics are shown at the score threshold of ≥2. The primary perioperative multivariable model included operative duration, intraoperative complication, COPD, and pre-existing arrhythmia; the preoperative-only model included COPD, pre-existing arrhythmia, FVC%, ASA physical status, and CCI. AUC, area under the receiver operating characteristic curve; CI, confidence interval; COPD, chronic obstructive pulmonary disease; NPV, negative predictive value; PPV, positive predictive value; Sn, sensitivity; Sp, specificity. (**B**) Note. Pairwise differences in AUC were evaluated using paired bootstrap resampling with 5000 resamples. AUC, area under the receiver operating characteristic curve; CI, confidence interval.

**Table 6:** (**A**). Observed actual postoperative ICU admission rates according to the exploratory perioperative triage score. (**B**). Classification performance of alternative exploratory perioperative ICU triage score thresholds.

(**A**)					
**Exploratory Perioperative ICU Triage Score**		**Total *n***		**Actual ICU Admission, *n* (%)**	
0		61		7 (11.5)	
1		58		14 (24.1)	
2		23		9 (39.1)	
3		14		12 (85.7)	
4		3		3 (100.0)	
(**B**)					
**Score Threshold**	**Sn**	**Sp**	**PPV**	**NPV**	**Accuracy**
≥1	0.844	0.474	0.388	0.885	0.579
≥2	0.533	0.860	0.600	0.824	0.767
≥3	0.333	0.982	0.882	0.789	0.799
≥4	0.067	1.000	1.000	0.731	0.736

(**A**) Note. The exploratory perioperative ICU triage score assigned 1 point for operative duration ≥180 min, 2 points for intraoperative complication, 1 point for COPD, and 1 point for pre-existing arrhythmia. A greater weight was assigned to intraoperative complication because it represented the strongest perioperative predictor in the primary model and had the largest relative clinical contribution to observed ICU admission. Although the theoretical maximum score was 5, no patient in the analytical cohort achieved a score of 5. The score 4 category included only three patients; estimates at this threshold should be interpreted with caution. COPD, chronic obstructive pulmonary disease; ICU, intensive care unit. (**B**) Note. Patients with a score equal to or above the stated threshold were classified as higher risk. The threshold of ≥2 points corresponded to the Youden-selected optimal cutoff within the present cohort and should be interpreted as exploratory rather than as a validated clinical decision threshold. ICU, intensive care unit; NPV, negative predictive value; PPV, positive predictive value; Sn, sensitivity; Sp, specificity.

## Data Availability

The data supporting the findings of this study are available from the corresponding author upon reasonable request and subject to institutional and ethical approval requirements. The data are not publicly available because they contain patient-level clinical information.

## Supplementary Materials

The following supporting information can be downloaded at: https://www.mdpi.com/article/10.3390/jcm15135043/s1, Supplementary Table S1: ROC analysis of operative duration for derivation of the binary threshold incorporated into the exploratory triage score; Supplementary Table S2: Variable completeness for key analytical variables; Supplementary Table S3: Sensitivity analysis of the primary model additionally including hypertension; Supplementary Table S4: Sensitivity analysis of the primary model additionally including estimated blood loss; Supplementary Table S5: Complete univariable logistic regression results for all evaluated candidate variables; Supplementary Table S6: Calibration and internal validation summary for the primary perioperative multivariable model; Supplementary Table S7: Components, definitions, and point assignment rationale for the exploratory perioperative ICU triage score; Supplementary Table S8: Baseline and intraoperative characteristics according to postoperative triage pathway; Supplementary File S1: Statistical analysis code used to reproduce the main analyses.

## Abbreviations

The following abbreviations are used in this manuscript:

ASA	American Society of Anesthesiologists
AUC	area under the receiver operating characteristic curve
CCI	Charlson Comorbidity Index
CI	confidence interval
COPD	chronic obstructive pulmonary disease
CRP	C-reactive protein
ECOG	Eastern Cooperative Oncology Group
ERS/ESTS	European Respiratory Society/European Society of Thoracic Surgeons
FEV_1_	forced expiratory volume in one second
FVC	forced vital capacity
ICU	intensive care unit
IQR	interquartile range
mFI-11	Modified Frailty Index-11
NPV	negative predictive value
NSCLC	non-small cell lung cancer
OR	odds ratio
PACU	post-anaesthesia care unit
PPV	positive predictive value
RATS	robot-assisted thoracoscopic surgery
ROC	receiver operating characteristic
Sn	sensitivity
Sp	specificity
SpO_2_	peripheral oxygen saturation
STROBE	Strengthening the Reporting of Observational Studies in Epidemiology
STS	Society of Thoracic Surgeons
TRIPOD	Transparent Reporting of a Multivariable Prediction Model for Individual Prognosis or Diagnosis
VATS	video-assisted thoracoscopic surgery
