# Supplementary material for: Refining Postoperative Intensive Care Triage After Anatomical Lung Resection: A Retrospective Cohort Study of Perioperative Reassessment
Source: J Clin Med. 2026 Jun 28;15(13):5043. doi: 10.3390/jcm15135043 (PMC13362921; doi:10.3390/jcm15135043)
Supplement: Supplementary file 1 [file jcm-15-05043-s001.zip › Supplementary Tables.pdf]

Supplementary Material

Supplementary Table S1. ROC analysis of operative duration for derivation of the binary threshold incorporated into the exploratory perioperative triage score.

Predictor	AUC (95% CI)	Youden-Selected Cutoff	Sensitivity	Specificity
Operative duration, min	0.709 (0.617–0.797)	≥180 min	0.467	0.868

Note. The cutoff was selected using the Youden index and incorporated into the exploratory perioperative triage score. Because it was derived and evaluated in the same cohort, it is exploratory. AUC, area under the receiver operating characteristic curve; CI, confidence interval; ICU, intensive care unit.

Supplementary Table S2. Variable completeness for key analytical variables.

Variable	Available n	Missing n	Missing (%)
Actual postoperative ICU admission	159	0	0.0
Operative duration	159	0	0.0
Intraoperative complication	159	0	0.0
COPD	159	0	0.0
Pre-existing arrhythmia	159	0	0.0
Hypertension	159	0	0.0
Estimated blood loss	159	0	0.0
FVC	159	0	0.0
ASA physical status	159	0	0.0
Charlson Comorbidity Index	159	0	0.0
C-reactive protein	159	0	0.0

Note. No missing values were identified for variables included in the primary model, comparator model, exploratory score, or prespecified sensitivity analyses in the 159-patient analytical cohort.

Supplementary Table S3. Sensitivity analysis of the primary model, including hypertension.

Variable	Adjusted OR (95% CI)	p Value
Operative duration, per minute	1.010 (1.004–1.017)	0.001
Intraoperative complication	12.923 (3.164–52.781)	<0.001
COPD	1.908 (0.834–4.366)	0.126
Pre-existing arrhythmia	2.511 (0.909–6.933)	0.076
Hypertension *	0.422 (0.177–1.002)	0.051

Note. * The inverse direction of the hypertension coefficient should not be interpreted as a biological protective effect in this selected cohort. CI, confidence interval; COPD, chronic obstructive pulmonary disease; OR, odds ratio.

Supplementary Table S4. Sensitivity analysis of the primary model, including estimated blood loss.

Variable	Adjusted OR (95% CI)	p Value
Operative duration, per minute	1.012 (1.005–1.018)	<0.001
Intraoperative complication	14.634 (3.014–71.051)	<0.001
COPD	1.994 (0.882–4.509)	0.097
Pre-existing arrhythmia	2.341 (0.862–6.359)	0.095
Estimated blood loss, per mL ‡	1.000 (0.997–1.003)	0.949

Note. ‡ Estimated blood loss was evaluated in sensitivity analysis only because of its markedly zero-inflated documentation pattern; a recorded value of 0 mL reflects the absence of separately documented clinically relevant blood loss rather than complete absence of bleeding. CI, confidence interval; COPD, chronic obstructive pulmonary disease; OR, odds ratio.

Supplementary Table S5. Complete univariable logistic regression results for all evaluated candidate variables.

Variable	Unadjusted OR (95% CI)	p Value
Patient Characteristics		
Age, per year	1.002 (0.968–1.038)	0.902
Body mass index, per kg/m²	0.976 (0.919–1.036)	0.420
Smoking exposure, per pack-year	1.001 (0.991–1.012)	0.811
Male sex	1.966 (0.829–4.660)	0.125
Pulmonary and Laboratory Variables		
FEV_1, per % predicted	0.996 (0.981–1.011)	0.612
FVC, per % predicted	0.988 (0.969–1.007)	0.226
FEV_1/FVC, per %	1.011 (0.988–1.034)	0.355
Preoperative SpO_2, per %	1.114 (0.953–1.301)	0.176
C-reactive protein, per mg/L	1.079 (0.909–1.282)	0.383
Clinical Status		
ASA physical status, per class	0.651 (0.330–1.282)	0.214
ECOG performance status, per point	0.888 (0.540–1.461)	0.640
Charlson Comorbidity Index, per point	0.929 (0.799–1.081)	0.342
Modified Frailty Index-11, per point	0.959 (0.044–21.042)	0.979
Comorbidities		
Hypertension	0.274 (0.129–0.585)	<0.001
Diabetes mellitus	0.730 (0.332–1.605)	0.434
COPD	1.959 (0.974–3.939)	0.059
Coronary artery disease	1.392 (0.694–2.794)	0.352
Pre-existing arrhythmia	2.135 (0.894–5.097)	0.087
Valvular heart disease	1.433 (0.626–3.281)	0.395
Previous coronary angioplasty/stent	1.521 (0.748–3.092)	0.247
Previous cerebrovascular event	1.014 (0.189–5.426)	0.987
Heart failure	0.644 (0.202–2.057)	0.458
Cardiology consultation	0.924 (0.463–1.842)	0.821
Operative Factors		
Operative duration, per minute	1.012 (1.006–1.018)	<0.001
Estimated blood loss, per mL ‡	1.004 (1.001–1.007)	0.014
Intraoperative complication	13.455 (3.582–50.542)	<0.001

Note. Variables affected by complete separation (vasopressor requirement) were not entered into logistic regression and are described separately in the main text. ‡ Estimated blood loss showed a markedly zero-inflated distribution; a recorded value of 0 mL indicated the absence of separately documented clinically relevant blood loss rather than complete absence of bleeding. Cardiology consultation was included in the complete univariable analysis for transparency but was subsequently removed from Table 1 following peer review because it reflects institutional referral practice rather than an intrinsic patient characteristic. ASA, American Society of Anesthesiologists; CCI, Charlson Comorbidity Index; CI, confidence interval; COPD, chronic obstructive pulmonary disease; ECOG, Eastern Cooperative Oncology Group; FEV_1, forced expiratory volume in one second; FEV_1/FVC, forced expiratory volume in one second to forced vital capacity ratio, expressed as a percentage; FVC, forced vital capacity; ICU, intensive care unit; OR, odds ratio; SpO_2, peripheral oxygen saturation.

Supplementary Table S6. Calibration and internal validation summary for the primary perioperative multivariable model.

Performance Measure	Value
Apparent AUC (primary model)	0.802
Brier score	0.147

Performance Measure	Value
Hosmer–Lemeshow χ^2 (df = 8)	12.659
Hosmer–Lemeshow p value	0.124
Bootstrap resamples requested	1000
Successful bootstrap resamples	983
Failed bootstrap resamples	17
Mean optimism	0.015
Optimism-corrected AUC	0.787

Note. Internal validation was performed using bootstrap optimism correction (1000 resamples); 17 resamples failed to converge owing to occasional complete or near-complete separation, and only the 983 successful model refits contributed to optimism estimation. Because the analytical cohort comprised 159 patients, each calibration decile contained approximately 15–16 observations; calibration estimates should therefore be interpreted as preliminary. A non-significant Hosmer–Lemeshow result in a small cohort does not constitute confirmation of good calibration. AUC, area under the receiver operating characteristic curve; df, degrees of freedom.

Supplementary Table S7. Derivation and point assignment of the exploratory perioperative ICU triage score.

Score component	Operational definition used in the score	Primary model estimate	Log coefficient and scaled weight	Point assignment rationale	Assigned points
Operative duration	≥180 min	Adjusted OR 1.012 per minute; 95% CI, 1.005–1.018; $p < 0.001$	β = 0.0115 per minute; scaled weight at 180 min = (0.0115 × 180 / 2.708) × 2 = 1.529; assigned 1 point pragmatically	Operative duration was dichotomised using the Youden-selected threshold of ≥180 min to improve bedside interpretability. Although the coefficient-based scaled weight was approximately 1.5, duration reflects a gradual exposure rather than a binary adverse intraoperative event; therefore, it was assigned 1 point for parsimony and bedside simplicity.	1
Intraoperative complication	Present	Adjusted OR 15.002; 95% CI, 3.738–60.210; $p < 0.001$	β = 2.708; reference strongest predictor; assigned 2 points	This was the strongest perioperative predictor in the primary model and represented an acute adverse intraoperative event. It was therefore used as the reference high-weight component and assigned 2 points.	2
COPD	Present	Adjusted OR 1.993; 95% CI, 0.882–4.507; $p = 0.097$	β = 0.690; scaled weight = (0.690 / 2.708) × 2 = 0.51; rounded to 1 point	COPD was retained as a clinically pre-specified baseline respiratory risk component. Its scaled contribution was smaller than intraoperative complication, but it was assigned 1 point to preserve clinical interpretability.	1
Pre-existing arrhythmia	Present	Adjusted OR 2.338; 95% CI, 0.861–6.351; $p = 0.096$	β = 0.849; scaled weight = (0.849 / 2.708) × 2 = 0.63; rounded to 1 point	Pre-existing arrhythmia was retained as a clinically pre-specified baseline cardiopulmonary risk component. Its scaled contribution was smaller than intraoperative complication, but it was assigned 1 point to preserve clinical interpretability.	1
Total score	Sum of assigned points	—	—	The final score range was calculated by summing component points.	0–5

Note. The exploratory perioperative ICU triage score was developed as a descriptive, hypothesis-generating risk stratification approach derived from the clinically pre-specified primary perioperative model. Operative duration was dichotomised at the Youden-selected threshold of ≥180 min. Integer points were assigned using a pragmatic combination of model-derived effect size, coefficient-based scaled weight, clinical interpretability, and bedside simplicity rather than as a formal validated nomogram. Intraoperative complication received 2 points because it had the largest adjusted effect estimate in the primary model and represented an acute intraoperative adverse event. Operative duration, COPD, and pre-existing arrhythmia were assigned 1 point each to preserve simplicity and interpretability. External validation is required before clinical use. COPD, chronic obstructive pulmonary disease; CI, confidence interval; ICU, intensive care unit; OR, odds ratio.

Supplementary Table S8. Baseline and intraoperative characteristics according to postoperative triage pathway.

Variable	No ICU admission (n = 114)	Direct ICU admission (n = 28)	PACU-to-ICU transfer (n = 17)	p value
Age, years	66.0 [60.2–71.0]	67.0 [60.8–72.2]	63.0 [56.0–72.0]	0.571
Body mass index, kg/m²	26.4 [23.7–30.1]	26.1 [23.6–28.9]	25.3 [24.0–27.1]	0.808
Smoking exposure, pack-years	40.0 [0.0–52.2]	40.0 [15.0–57.0]	30.0 [20.0–40.0]	0.719
FEV_1, % predicted	79.5 [60.2–96.0]	74.0 [63.0–84.0]	78.0 [53.0–101.0]	0.864
FVC, % predicted	78.5 [65.5–91.0]	73.5 [64.5–84.8]	70.0 [64.0–92.0]	0.458
FEV_1/FVC, %	106.0 [95.0–114.0]	107.0 [97.8–114.0]	109.0 [101.0–116.0]	0.687
ASA physical status	3.0 [3.0–3.0]	3.0 [2.0–3.0]	2.0 [2.0–3.0]	0.018
ECOG performance status	2.0 [1.0–2.0]	1.0 [1.0–2.0]	1.0 [1.0–2.0]	0.505
Preoperative SpO_2, %	96.0 [95.0–97.8]	97.0 [95.0–98.0]	97.0 [93.0–98.0]	0.081
Modified Frailty Index-11	0.2 [0.1–0.3]	0.2 [0.1–0.2]	0.2 [0.1–0.3]	0.794
Charlson Comorbidity Index	7.0 [5.0–8.0]	6.0 [5.0–8.0]	6.0 [5.0–7.0]	0.623
Male sex	80 (70.2)	24 (85.7)	13 (76.5)	0.238
Hypertension	65 (57.0)	5 (17.9)	7 (41.2)	<0.001
Diabetes mellitus	35 (30.7)	5 (17.9)	6 (35.3)	0.336
COPD	42 (36.8)	16 (57.1)	8 (47.1)	0.132
Pre-existing arrhythmia	15 (13.2)	7 (25.0)	4 (23.5)	0.221
Coronary artery disease	44 (38.6)	12 (42.9)	9 (52.9)	0.518
Heart failure	15 (13.2)	2 (7.1)	2 (11.8)	0.679
Operative duration, min	95.0 [70.0–140.0]	180.0 [130.0–222.5]	110.0 [70.0–180.0]	<0.001
Estimated blood loss, mL	0.0 [0.0–0.0]	0.0 [0.0–25.0]	0.0 [0.0–0.0]	<0.001
Intraoperative complication	3 (2.6)	9 (32.1)	3 (17.6)	<0.001
Vasopressor requirement	0 (0.0)	6 (21.4)	0 (0.0)	<0.001
Invasive monitoring	0 (0.0)	28 (100.0)	16 (94.1)	<0.001

Note. Data are presented as median [interquartile range] or number (%), as appropriate. Continuous variables were compared using the Kruskal–Wallis test. Categorical variables were compared using Pearson's chi-square test. Estimated blood loss showed a markedly zero-inflated distribution; recorded values of 0 mL indicated the absence of separately documented clinically relevant blood loss rather than complete absence of bleeding. Invasive monitoring was included for descriptive comparison only and was not considered a candidate predictor because its use reflected clinician risk assessment and anticipated postoperative disposition. ASA, American Society of Anesthesiologists; COPD, chronic obstructive pulmonary disease; ECOG, Eastern Cooperative Oncology Group; FEV_1, forced expiratory volume in one second; FVC, forced vital capacity; ICU, intensive care unit; PACU, post-anaesthesia care unit; SpO_2, peripheral oxygen saturation.
